# Freestanding Hierarchical‐Porous BCZT Thin Films Enable Efficient Ultrasonic Energy Harvesting in Soft Tissue

**DOI:** 10.1002/advs.77409

**Published:** 2026-08-29

**Authors:** Zifan Li, Shengwei Gao, Wang Hong, Yuxin Chen, Yiyang Gu, Xu Wang, Chen Tang, Ying Hong, Xuemu Li, Xiaodong Yan, Yi Zheng, Yiming Liu, Junchen Liao, Biao Wang, Shiyuan Liu

**Affiliations:** ^1^ Thrust of Smart Manufacturing System Hub The Hong Kong University of Science and Technology (Guangzhou) Guangzhou China; ^2^ Institute of Advanced Structure Technology Beijing Institute of Technology Beijing China; ^3^ Department of Medical Ultrasound National Clinical Research Center for Geriatrics West China Hospital Sichuan University Chengdu China; ^4^ Department of Mechanical and Aerospace Engineering The Hong Kong University of Science and Technology Hong Kong Kowloon China; ^5^ Guangdong Fenghua Advanced Technology Holding Co., Ltd Zhaoqing Guangdong China; ^6^ Macao Institute of Materials Science and Engineering (MIMSE) Faculty of Innovation Engineering Macau University of Science and Technology Taipa Macao China; ^7^ Department of Biomedical Engineering City University of Hong Kong Hong Kong Kowloon China

**Keywords:** BCZT thin film, hierarchical‐porous, stress dispersion, ultrasound energy harvesting, wireless wearable electronics

## Abstract

Excellent electromechanical properties of piezoceramic thin films facilitate ultrasonic energy harvesting in bioelectronic systems. However, most piezoceramic thin films are fragile and cannot maintain mechanical integrity under deformation, hindering their application in soft tissue coupling. Here, we report a freestanding hierarchical‐porous barium calcium zirconate titanate (BCZT) thin film that combines crack resistance with efficient ultrasonic energy harvesting. The hierarchical‐porous structure, fabricated using polyvinylpyrrolidone (PVP) as a porogen, disperses tensile‐side stress concentration and suppresses crack initiation, while reducing the acoustic‐impedance mismatch with soft tissue and suppressing interfacial ultrasound reflection. The film exhibits reduced permittivity and a high piezoelectric voltage coefficient of g_33_ = 97 × 10^−3^ V·m/N, resulting in a maximum output power that is approximately 1.5 times that of the dense BCZT thin film under identical ultrasound excitation, evaluated at the respective optimal loads. Owing to its excellent piezoelectric performance, the film also enables reliable detection of weak vibrations, such as radial artery pulse‐wave signals. This work demonstrates a hierarchical‐porous structure for simultaneously improving acoustic coupling, mechanical reliability, and electromechanical transduction in freestanding piezoceramic thin films, highlighting their potential for wearable sensing and implantable ultrasound‐powered bioelectronics.

## Graphical abstract

Freestanding hierarchical‐porous BCZT thin films integrate a compliant porous surface with a dense piezoceramic backbone to balance mechanical reliability, voltage sensitivity, and acoustic coupling. The films enable stable wearable pulse‐wave monitoring and enhanced tissue‐coupled ultrasonic energy harvesting, offering a promising route toward soft biointegrated sensors and ultrasound‐powered implantable electronics.

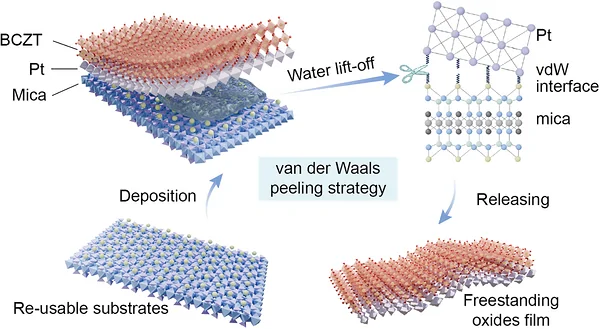

## 1 Introduction

Piezoelectric thin films play a central role in flexible sensing [1, 2, 3, 4, 5, 6, 7] and actuation [8], owing to their intrinsic compatibility with device miniaturization and system integration. However, most piezoceramic thin films are deposited and processed on rigid substrates, and the resulting mechanical constraint severely limits their deformability and conformal integration with soft, dynamically moving tissues [9, 10]. This limitation hinders their use in flexible electronics and implantable bioelectronics that require intimate tissue contact and compliance under bending, twisting, and micromotions [11, 12]. Thus, developing freestanding piezoelectric ceramic thin films with excellent mechanical reliability is essential for practical advancements.

In recent years, various strategies for fabricating freestanding piezoelectric ceramic thin films have emerged, including chemical etching [13, 14, 15], mechanical peeling [16, 17], and laser lift‐off methods [18]. These approaches enable effective release of the films from rigid substrates, while largely preserving their intrinsic piezoelectric performance. However, piezoelectric ceramic thin films exhibit inherent brittleness, which makes the peeling process highly susceptible to mechanical damage, including cracking and tearing [19, 20]. This not only poses challenges for fabricating large‐area, intact freestanding films, but also severely restricts their practical application in flexible sensors. Moreover, when such freestanding piezoceramic thin films are used in flexible sensors, stress tends to concentrate in the ceramic layer during device deformation, making it prone to cracking. These cracks can lead to electrical short circuits between the top and bottom electrodes, ultimately resulting in complete device failure [21, 22].

To enable low‐damage release of piezoelectric ceramic thin films, a van der Waals weak‐interface strategy based on Pt/mica heterojunctions provides an effective route for freestanding‐film fabrication [9, 23, 24]. In this process, water infiltrates from the film edge and propagates along the weak Pt/mica interface through capillary action, allowing mild and controllable delamination while reducing the mechanical damage associated with conventional peeling. However, successful release alone does not fully solve the mechanical reliability issue. Freestanding piezoceramic films remain intrinsically brittle during subsequent device integration and operation, where localized tensile stress can easily develop under bending and tissue‐induced deformation. Introducing pores into dense ceramics can improve structural compliance and mitigate stress concentration, but excessive or uniformly distributed porosity may weaken the ceramic backbone and compromise mechanical integrity [25, 26, 27]. Therefore, a hierarchical‐porous architecture is introduced to balance stress redistribution and structural support. The porous region accommodates deformation through local pore‐wall distortion and network‐level compliance, while the relatively dense region preserves ceramic continuity and mechanical robustness [27].

In addition to mechanical reliability, implantable ultrasonic energy harvesting also requires piezoceramic thin films to provide high voltage sensitivity and efficient acoustic coupling with soft tissue. A high dielectric permittivity can suppress the voltage response, whereas the large acoustic‐impedance mismatch between dense ceramics and soft biological tissues causes interfacial reflection and reduces ultrasound transmission into the active piezoelectric layer [12, 28, 29]. These limitations motivate the design of a freestanding hierarchical‐porous barium calcium zirconate titanate (BCZT) thin film with a porous tissue‐contacting surface gradually transitioning toward a denser ceramic backbone. This architecture is designed to reduce the effective dielectric permittivity, maintain appreciable piezoelectric activity, and provide a gradual acoustic‐impedance transition at the tissue‐film interface. As a result, the same hierarchical‐porous design integrates deformation accommodation, voltage sensitivity, and tissue‐coupled acoustic transmission, enabling wearable pulse‐wave sensing and implantable ultrasound‐mediated energy delivery.

## 2 Results

### 2.1 Fabrication of Freestanding Hierarchical‐Porous BCZT Thin Films

**FIGURE 1:**
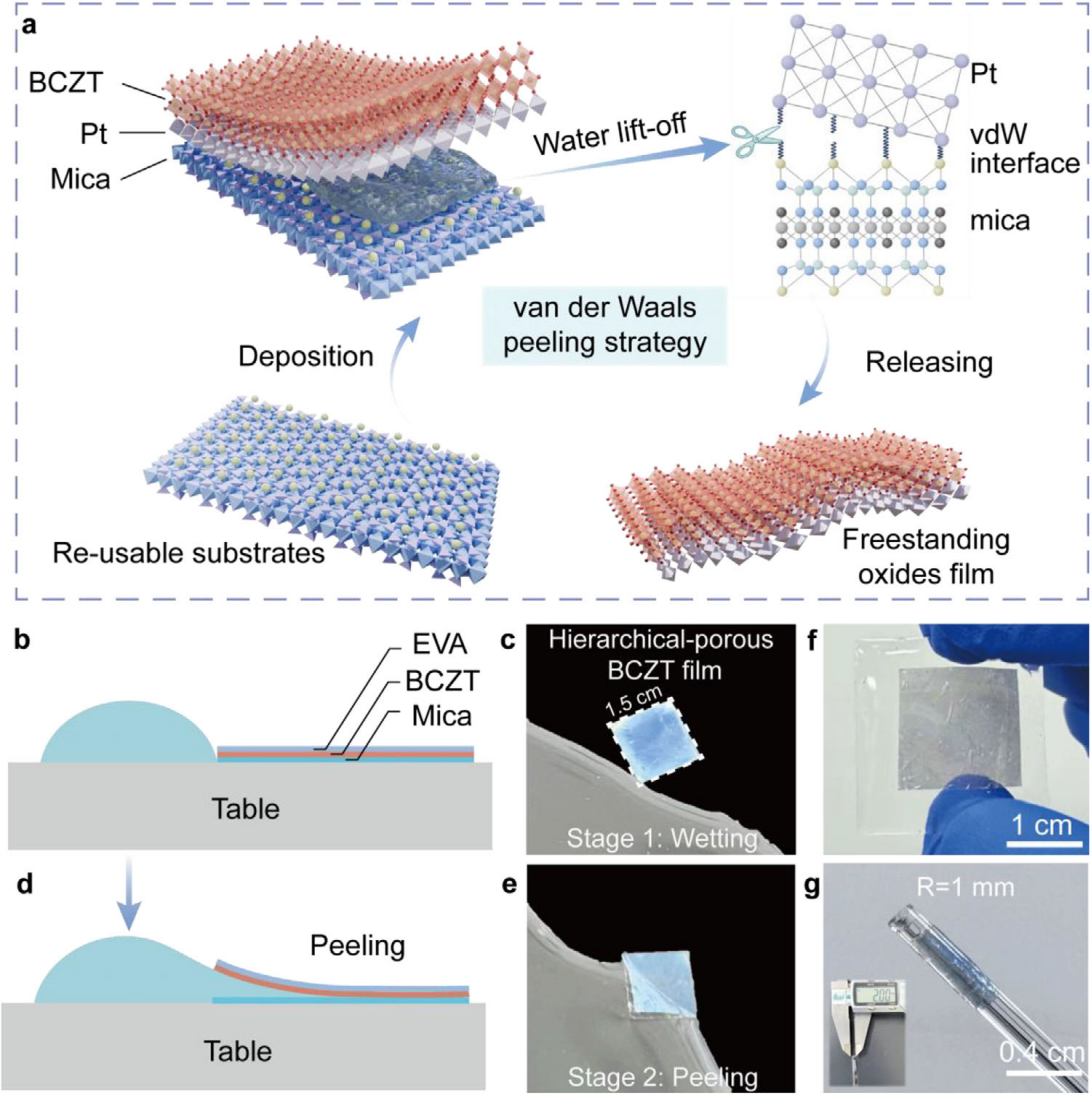
Water‐triggered van der Waals lift‐off and flexibility of freestanding hierarchical‐porous BCZT thin films. (a) Schematic of deposition on Pt/mica and water‐assisted release at the Pt/mica van der Waals interface. (b,d) Side‐view schematics of wetting‐assisted peeling and progressive peeling. (c,e) Photographs showing Stage 1 (wetting) and Stage 2 (peeling). (f) Photograph of the released freestanding hierarchical‐porous BCZT film. (g) Bending demonstration of the released film at R ≈ 1 mm.

To fabricate freestanding hierarchical‐porous BCZT thin films while minimizing release‐induced damage, we developed a water‐triggered van der Waals lift‐off strategy based on a Pt‐coated mica substrate (Figure 1a). In this design, the buried Pt/mica interface serves as a weak adhesion layer that enables controlled film release while avoiding the large mechanical loads typically imposed during conventional peeling. This strategy therefore helps preserve the structural integrity of the brittle piezoceramic membrane during delamination.

The hierarchical‐porous BCZT film was first deposited on the Pt‐coated mica substrate and then covered with an ethylene‐vinyl acetate (EVA) support layer (Figure 1a). The EVA layer mechanically stabilizes the fragile ceramic membrane during release and transfer. Water was introduced from the sample edge to initiate interfacial separation. Once the liquid penetrated the buried Pt/mica interface, delamination propagated along this weak van der Waals interface rather than through fracture of the ceramic layer, enabling controlled release of the BCZT/Pt stack from the mica substrate. After wetting, water gradually infiltrated the Pt/mica interface and progressively lifted the EVA/BCZT/Pt stack away from the substrate, as reflected by the side‐view schematics of interfacial wetting and progressive delamination (Figure 1b,d) and the corresponding optical photographs capturing the transition from the intact supported state to the partially released state (Figure 1c,e). This gradual interfacial propagation mitigated abrupt local stress concentration and thereby reduced the likelihood of crack formation in the ceramic layer, confirming the feasibility of the water‐triggered lift‐off route.

To clarify the delamination plane during water‐triggered lift‐off, large‐area scanning electron microscopy (SEM) coupled with energy‐dispersive X‐ray spectroscopy (EDS) mapping was performed on the exposed backside of the released BCZT/Pt film, corresponding to the Pt‐side surface after lift‐off. The mapping was conducted over a millimeter‐scale region to avoid drawing conclusions from a localized point measurement (Figure S1). A continuous Pt signal was observed over the mapped region, confirming that the exposed backside corresponds to the Pt electrode layer. Weak Ba, Ca, Ti, and O signals were attributed to the underlying BCZT layer because of the finite EDS interaction volume through the thin Pt electrode. No detectable mica‐related elements, such as Al, Mg, K, or Fe, were observed in the EDS spectrum or elemental quantification. Although a strong Si signal was detected, it originated from the Si wafer used as the supporting substrate during SEM‐EDS characterization. These results indicate that no detectable mica residue remains on the exposed Pt‐side surface and support that water‐triggered delamination occurs predominantly at the Pt/mica interface rather than through intralayer cleavage within mica.

The reusability of the mica substrate was also examined by performing two successive deposition‐release cycles using the same mica substrate (Figure S2). Photographs show that the BCZT/Pt film could be released from the mica substrate through the water‐triggered lift‐off process in both cycles. This result demonstrates that the mica substrate can be reused for at least two deposition‐release cycles while maintaining the feasibility of water‐triggered lift‐off.

After release, an intact freestanding membrane with a centimeter‐scale lateral size was obtained (Figure 1f). The film retained its overall geometry without visible fragmentation, indicating that the combination of the weak Pt/mica interface and EVA‐assisted transfer effectively protected the porous BCZT layer during release. The surface integrity of the transferred BCZT film after EVA removal was further examined by atomic force microscopy (AFM) and SEM. AFM height imaging and 3D topography reveal a continuous surface morphology without long trench‐like cracks, sharp step‐like discontinuities, or obvious transfer‐induced fracture features within the scanned region (Figure S3). The observed height variation is mainly associated with the intrinsic surface roughness of the porous ceramic film rather than macroscopic cracking. Large‐area SEM observation further confirms that no obvious crack networks, through‐film cracks, or fragmentation features are observed after EVA removal. These observations indicate that the EVA‐assisted transfer and subsequent EVA removal process preserve the mechanical and microstructural integrity of the BCZT film within the characterized regions. This integrity is particularly important for freestanding ceramic thin films, which are otherwise highly susceptible to tearing or fracture after detachment from rigid substrates.

The released hierarchical‐porous BCZT film also exhibited pronounced mechanical deformability. It could be bent to a radius of approximately 1 mm without visible cracking (Figure 1g), demonstrating its ability to withstand large‐curvature deformation. Such compliance is difficult to achieve in conventional dense ceramic films, where tensile stress typically localizes during bending and readily triggers fracture. By contrast, the hierarchical‐porous architecture introduces additional structural compliance, allowing strain redistribution within the film and thereby improving bending tolerance.

### 2.2 Hierarchical‐Porous Structure and Stress Redistribution

**FIGURE 2:**
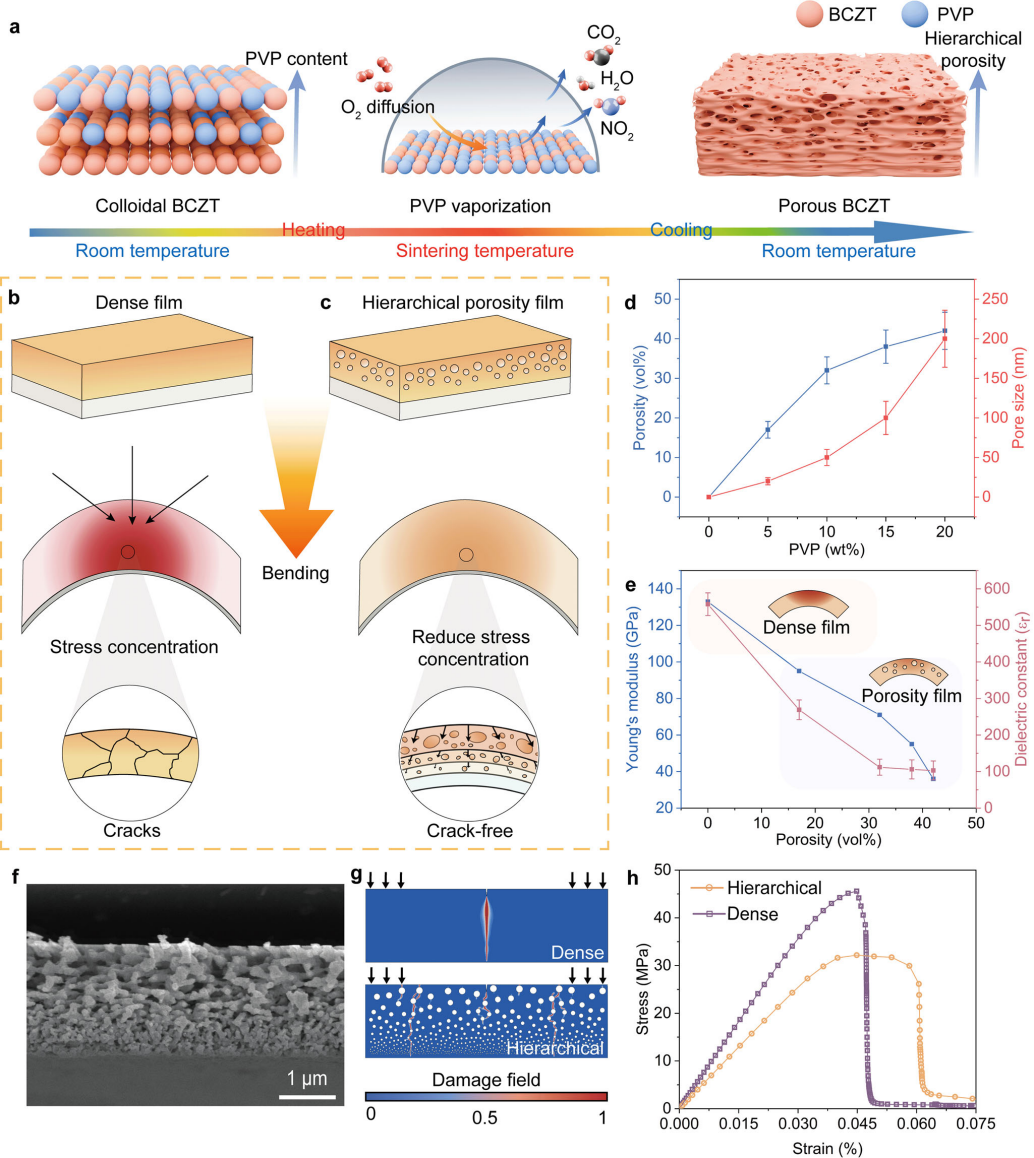
Formation of hierarchical porosity and its influence on bending mechanics in freestanding BCZT thin films. (a) Schematic illustration of PVP‐assisted pore generation and hierarchical porosity formation during heating. (b,c) Schematic comparison of stress concentration in dense and hierarchical‐porous films under bending. (d) Porosity and average pore size of BCZT thin films prepared with different PVP contents. (e) Porosity‐dependent Young's modulus and dielectric constant of BCZT thin films. (f) Cross‐sectional SEM image of the hierarchical‐porous BCZT film. (g) Phase‐field simulated damage patterns in dense and hierarchical films. (h) Stress‐strain response of dense and hierarchical films.

To understand the formation of the hierarchical‐porous architecture and its contribution to the mechanical reliability of freestanding BCZT thin films, we first investigated the thermal evolution of the polyvinylpyrrolidone (PVP)‐containing precursor film. As illustrated schematically, the hierarchical‐porous framework arises from the thermally induced removal and oxidation of PVP (Figure 2a). The direct removal of PVP generates small pores at its original locations, whereas the oxidation of the organic phase releases gaseous species whose outward diffusion enlarges the pore network and produces larger voids. Because the film was deposited layer by layer with progressively increased PVP content through the thickness, pore formation does not occur uniformly but instead develops into a graded porous‐to‐dense structure with a more porous upper region and a denser ceramic backbone beneath. The as‐deposited film was first subjected to a slow pre‐carbonization treatment under Ar, which moderates polymer decomposition and helps preserve structural continuity during the initial stage of pore formation (Figure S4). The pre‐carbonized film was subsequently annealed in air to remove residual carbonaceous species and induce BCZT crystallization (Figure S5). X‐ray diffraction (XRD) analysis shows that the film annealed at 700°C remains insufficiently crystallized and still exhibits a residual carbon‐related feature, whereas well‐defined BCZT diffraction peaks emerge after annealing at 800°C and 900°C, indicating successful phase formation at higher temperatures (Figure S6). Cross‐sectional SEM images further reveal that these annealing temperatures do not preserve the porous framework equally well. The film annealed at 800°C retains a well‐defined porous structure, whereas partial pore collapse is observed after annealing at 900°C because of excessive sintering and densification (Figure S7). Together, these results identify 800°C as the optimal thermal window for constructing the hierarchical‐porous BCZT architecture, because it enables effective organic removal and crystallization while preserving the porous framework.

Such a hierarchical‐porous architecture is expected to provide a viable route to mitigate bending‐induced stress concentration in freestanding BCZT thin films. Whereas a dense ceramic film generally develops a localized tensile stress hotspot at the outer surface during bending, a porous network is anticipated to accommodate deformation through local pore‐wall distortion and network‐level compliance, thereby distributing stress more broadly across the film (Figure 2b,c). On this basis, we next designed and analyzed a hierarchical‐porous configuration that could redistribute bending‐induced stress while maintaining sufficient film integrity.

To quantitatively establish the relationship between PVP loading and the resulting pore structure, BCZT films with different PVP contents were further prepared and analyzed. The influence of PVP loading on pore morphology and porosity was first examined. Surface and cross‐sectional SEM images show that increasing the PVP content progressively increases the pore fraction and drives the microstructure from isolated pores toward a more interconnected porous network (Figure S8). Quantitative image analysis further shows that both the porosity and average pore size increase systematically with increasing PVP content (Figure 2d). The porosity increases from nearly 0 vol% for the dense film to approximately 17, 32, 38, and 42 vol% for the films prepared with 5, 10, 15, and 20 wt.% PVP, respectively. Meanwhile, the average pore size also increases monotonically with PVP loading, confirming the effective pore‐forming role of PVP.

Since porosity is the direct microstructural feature governing the effective properties of porous ceramics, the material properties were further analyzed as functions of porosity. Both the Young's modulus and dielectric constant decrease with increasing porosity (Figure 2e). The reduction in Young's modulus indicates that the porous framework progressively softens the BCZT film and enhances structural compliance. The decrease in dielectric constant can be attributed to the replacement of part of the high‐permittivity BCZT ceramic phase by low‐permittivity air voids. This dielectric reduction is beneficial for improving the piezoelectric voltage coefficient g_33_, whereas excessive porosity may weaken the ceramic backbone and reduce effective poling efficiency. Therefore, the PVP content and porosity must be selected within an appropriate range to balance dielectric reduction, mechanical compliance, and piezoelectric retention.

Based on this structure‐property relationship, a three‐layer architecture with PVP contents of 0 wt.% in the bottom layer, 5 wt.% in the middle layer, and 10 wt.% in the top layer was adopted. This stepwise design establishes a graded porous‐to‐dense structure, in which the relatively dense bottom layer preserves mechanical support and piezoelectric continuity, while the more porous upper layers provide enhanced compliance for stress accommodation. The resulting through‐thickness hierarchical microstructure is confirmed by the cross‐sectional SEM image of the prepared film (Figure 2f). Consistent with this design rationale, the integrated flexible device exhibited distributed deformation under both bending and twisting, as evidenced by the experimental photographs and the corresponding finite‐element simulations (Note S1). Under these loading modes, the stress was not confined to a localized hotspot but instead spread more broadly throughout the device, indicating that the modulus‐graded hierarchical‐porous BCZT layer effectively mitigates stress concentration and preserves structural continuity at the device level (Figure S9).

To further evaluate how the hierarchical‐porous architecture influences fracture behavior, we performed phase‐field simulations based on the experimentally observed pore‐size distribution and porosity (Note S2 and Table S1). The hierarchical‐porous model contained pores with diameters ranging from 10 to 150 nm and an overall porosity of approximately 30 vol%, consistent with the cross‐sectional SEM observations. The final damage field shows a clear difference between the hierarchical‐porous and dense structures (Figure 2g), while the full damage evolution process is provided (Figure S10). In the dense ceramic, damage remains highly localized and rapidly develops into a dominant crack. In contrast, the hierarchical‐porous structure exhibits a more distributed damage pattern accompanied by crack deflection and branching. Multiple crack‐active regions appear within the porous structure, increasing the crack‐propagation path and promoting stress redistribution during bending. The corresponding mechanical response was plotted as stress–strain curves to evaluate the deformation behavior (Figure 2h). Compared with the dense film, the hierarchical‐porous film exhibits a lower failure stress but sustains a larger failure strain before final failure. To further quantify the mechanical response, the area under the stress‐strain curve was calculated as the mechanical energy density absorbed before failure.

The hierarchical‐porous film exhibited an energy density to failure of 0.014 MJ/m^3^, slightly higher than that of the dense film, 0.013 MJ/m^3^. This modest increase does not indicate a substantial enhancement in the intrinsic fracture toughness of the ceramic film. Instead, its mechanical benefit mainly arises from stress redistribution, crack deflection, and the ability to sustain a larger failure strain under the designed bending condition.

### 2.3 Electromechanical Characterization and Wearable Pulse‐Wave Sensing

**FIGURE 3:**
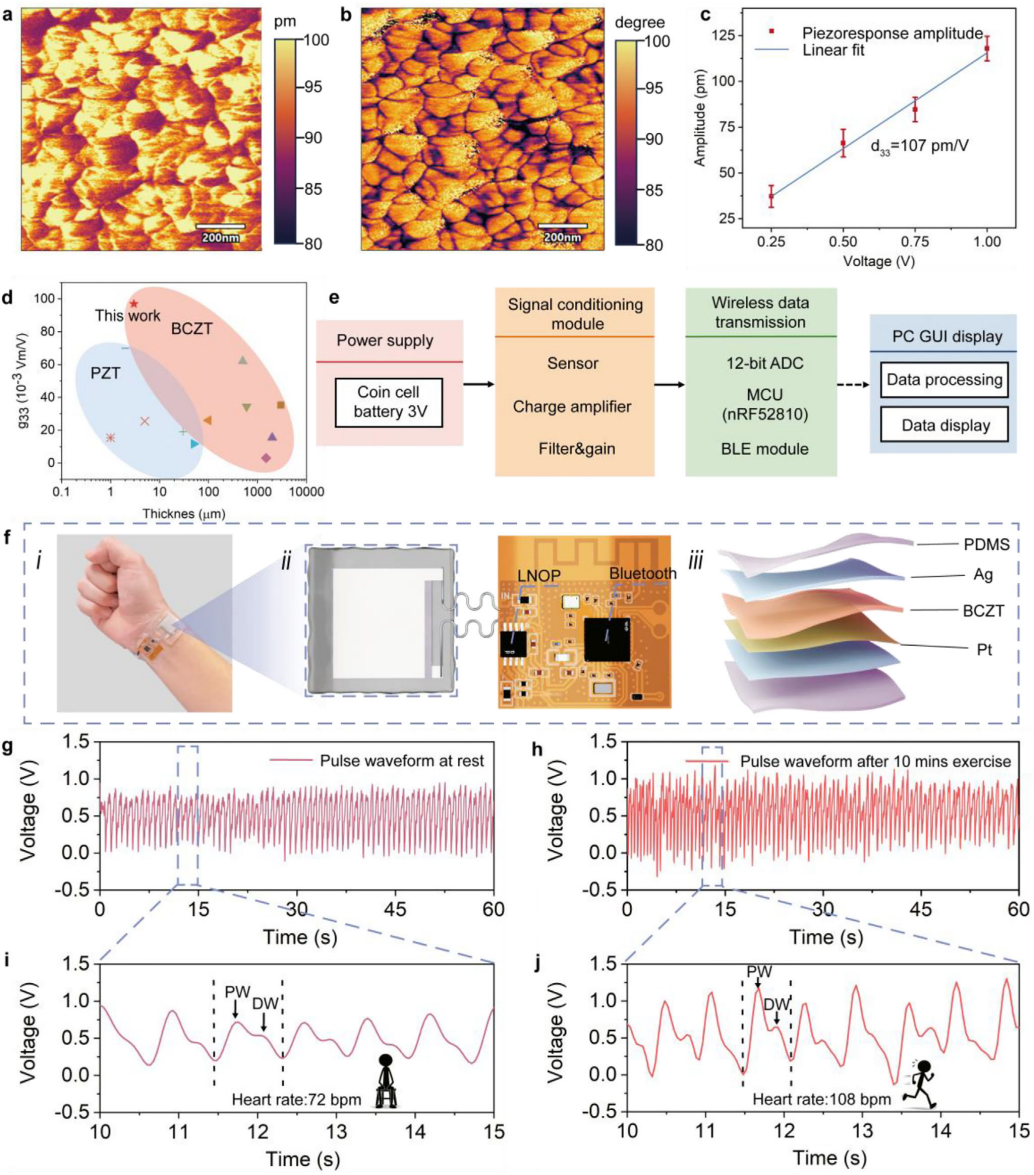
Electromechanical characterization and wearable pulse‐wave sensing based on hierarchical‐porous BCZT thin films. (a,b) PFM amplitude (Vac = 1 V) and phase maps. (c) PFM amplitude versus drive voltage with linear fitting. (d) Calculated g_33_ benchmarked against lead zirconate titanate (PZT) and reported BCZT [30, 31, 32, 33, 34, 35, 36, 37, 38, 39, 40, 41, 42, 43, 44, 45, 46]. (e) Schematic of the signal‐conditioning and wireless readout circuit. (f) i, Photograph of the wearable device on the wrist. ii) Photograph of the packaged sensing unit and flexible readout circuit. iii) Exploded view of the device architecture. (g,h) Pulse‐wave signals recorded at rest and after exercise. (i,j) Enlarged representative pulse‐wave cycles.

After release and corona poling, the hierarchical‐porous BCZT thin film was first evaluated at the microscale to assess the uniformity of its local electromechanical activity for device‐level sensing. Piezoresponse force microscopy (PFM) measurements showed that the film maintained a well‐defined piezoelectric response across the scanned region. The amplitude map recorded under a 1 V alternating‐current (AC) excitation displayed a relatively homogeneous contrast without obvious inactive domains or severely weakened regions, indicating that the electromechanical response was spatially continuous over the probed surface (Figure 3a). The corresponding phase image exhibited only limited local variation, suggesting that the ferroelectric domains were predominantly aligned after poling (Figure 3b). Such a uniform polarization state is important because it minimizes cancellation among differently oriented domains and supports a stable macroscopic voltage output from the freestanding film.

To quantify the local piezoelectric response, the dependence of the PFM amplitude on the applied AC drive voltage was measured. The piezoresponse of the hierarchical‐porous BCZT film increased nearly linearly as the drive voltage was raised from 0.25 to 1.0 V, and the fitted slope yielded a PFM‐derived effective d_33_ value of approximately 107 pm/V (Figure 3c). This value confirms that the hierarchical‐porous film retains appreciable local piezoelectric activity after release, transfer, and corona poling.

The effective piezoelectric response of the hierarchical‐porous film was further compared with that of the dense BCZT thin film. Under the same PFM measurement conditions, the dense film exhibited a higher effective d_33_ value of approximately 193 pm/V (Figure S11). This difference is reasonable because the dense film contains larger grains and a more continuous ceramic network, which are beneficial for domain switching and piezoelectric deformation. In contrast, the hierarchical‐porous film contains smaller grains and a porous ceramic framework. Higher‐magnification SEM images show that the grains in the hierarchical‐porous film are generally smaller than those in the dense film. This grain‐size reduction is attributed to the PVP‐derived porous network, which decreases the effective contact area between neighboring ceramic grains and hinders mass transport during sintering. In addition, the gradient distribution of PVP content produces through‐thickness variations in local pore fraction and pore connectivity, leading to non‐uniform grain development across the hierarchical‐porous film. Therefore, the lower effective d_33_ value of the hierarchical‐porous film should be understood as arising from the combined effects of smaller grain size, reduced ceramic connectivity, and decreased active ceramic volume, rather than from porosity alone.

To further examine whether the difference in effective piezoelectric response is also related to poling efficiency, PFM switching spectroscopy was performed on both dense and hierarchical‐porous BCZT thin films. Both films exhibit clear phase‐voltage hysteresis and butterfly‐shaped strain‐voltage responses, confirming switchable local piezoresponse after corona poling (Figure S12). Based on the switching voltage extracted from the PFM phase/strain curves and the film thickness, the apparent local coercive field was estimated to be approximately 2.4 × 10^6^ V/m. This value was used as a reference threshold to evaluate whether the local electric field in the ceramic phase during poling was sufficient to induce polarization switching. Electrostatic simulations were then performed to analyze the local electric‐field distribution during poling. Under the simulated through‐thickness poling condition, the dense BCZT film exhibits a relatively uniform field distribution, and most regions experience an electric field higher than the PFM‐estimated apparent coercive field (Figure S13 and Note S3). In contrast, the hierarchical‐porous film shows a more non‐uniform electric‐field distribution because of the large dielectric contrast between the BCZT ceramic phase and air voids. Local field concentration appears near the pore edges, while the void regions themselves cannot be polarized. Nevertheless, except for the highly porous surface region and local regions adjacent to large pores, most of the continuous ceramic skeleton still experiences an electric field comparable to or higher than the apparent coercive field. This result indicates that the ceramic framework of the hierarchical‐porous film can still be effectively polarized. However, compared with the dense film, the hierarchical‐porous film has a smaller effective polarizable ceramic volume because the air voids do not contribute to ferroelectric polarization. In addition, local field redistribution around pores lowers the poling uniformity in some ceramic regions. Therefore, in addition to smaller grain size and reduced ceramic connectivity, the reduced effective polarizable volume and non‐uniform local poling field also contribute to the lower effective d_33_ value of the hierarchical‐porous film compared with the dense film.

Although the hierarchical‐porous film shows a lower effective d_33_ than the dense film, the voltage‐generation capability of a piezoelectric film is governed not only by d_33_, but also by its dielectric permittivity. Therefore, the piezoelectric voltage coefficient g_33_ was calculated according to g_33_ = d_33_/(ε_0_ε_r_). The dense BCZT film exhibited a relatively high dielectric constant of approximately 560, which gives a calculated g_33_ value of approximately 39 × 10^−3^ V·m/N. In contrast, the hierarchical‐porous BCZT film showed a much lower dielectric constant of approximately 124 because part of the high‐permittivity ceramic phase was replaced by low‐permittivity air voids. Combined with the PFM‐derived effective d_33_ value of approximately 107 pm/V, the hierarchical‐porous film yielded a higher g_33_ value of 97 × 10^−3^ V·m/N, which is approximately 2.5 times that of the dense film (Figure 3d). This comparison indicates that the hierarchical‐porous structure sacrifices part of the effective d_33_ response, but the pronounced reduction in dielectric permittivity leads to a substantially enhanced voltage coefficient. Therefore, the advantage of the hierarchical‐porous BCZT film lies not in maximizing d_33_, but in achieving high voltage sensitivity for pulse‐wave sensing and ultrasonic energy harvesting.

The metal‐insulator‐metal (MIM) sandwich electrode configuration was selected to match the working mode of pulse‐wave sensing. During radial artery pulse monitoring, the device mainly experiences out‐of‐plane pressure and slight bending deformation induced by arterial pulsation. The MIM structure aligns the polarization direction, mechanical excitation direction, and charge‐collection direction along the film thickness, enabling efficient d_33_‐mode signal generation. In addition, the continuous top and bottom electrodes collect charges over the full active area, which is beneficial for detecting weak pulse‐induced signals and achieving stable pulse‐wave acquisition.

Based on this combination of high voltage sensitivity and deformation accommodation capability, the hierarchical‐porous BCZT film was further integrated into a wearable pulse‐wave sensing system. Because the electrical signal generated by the thin piezoelectric film under arterial pulsation is relatively weak and the device behaves as a high‐impedance source, dedicated signal conditioning was required before wireless acquisition. A custom flexible printed circuit board was therefore designed to enable stable signal readout and transmission (Figure 3e). The circuit integrates a charge‐amplifier‐based front end, a filtering and gain stage, a 12‐bit analog‐to‐digital converter (ADC), and a Bluetooth Low Energy (BLE) transmission module controlled by a microcontroller unit (MCU). In this configuration, the charge amplifier converts the charge output of the sensor into a voltage signal while minimizing loading effects, and the subsequent analog conditioning suppresses baseline drift and noise before digitization. The processed signal is then transmitted wirelessly for real‐time display and storage, providing a compact platform for wearable pulse‐wave monitoring.

To implement the sensing unit in a wearable format, the hierarchical‐porous BCZT membrane was integrated into a laminated flexible device structure (Figure 3f). The assembled sensor consists of polydimethylsiloxane (PDMS) encapsulation layers, Ag and Pt electrodes, and the freestanding BCZT active layer. In this design, the metallic electrodes enable stable charge collection from the piezoelectric layer, whereas the soft PDMS layers improve handling robustness, protect the ceramic film from direct mechanical damage, and maintain conformal contact with curved skin surfaces. The asymmetric encapsulation further mitigates the adverse influence of neutral‐plane positioning on the active ceramic layer, thereby preserving effective strain transfer during bending and skin deformation.

The wearable sensing performance of the hierarchical‐porous BCZT device was evaluated by monitoring wrist pulse waves under resting and post‐exercise conditions (Movie S1). Stable pulsatile voltage signals were clearly recorded in both states, demonstrating that the device can reliably capture weak arterial motions under practical wearing conditions (Figure 3g,h). Compared with the resting state, the post‐exercise waveform exhibited a shorter temporal interval between adjacent pulses, consistent with an increased heart rate after 10 min of exercise. Enlarged views of representative pulse cycles further resolved characteristic pulse‐wave features, including the percussion wave and dicrotic wave, in both states (Figure 3i,j). The extracted heart rates were approximately 72 beats per minute (bpm) at rest and 108 bpm after exercise, confirming that the freestanding hierarchical‐porous BCZT film can sensitively transduce physiological pulsation into readable electrical signals. These results demonstrate the potential of the device for wearable pulse‐wave monitoring and physiological state tracking.

### 2.4 Tissue‐Coupled Ultrasonic Energy Harvesting and Power Delivery

**FIGURE 4:**
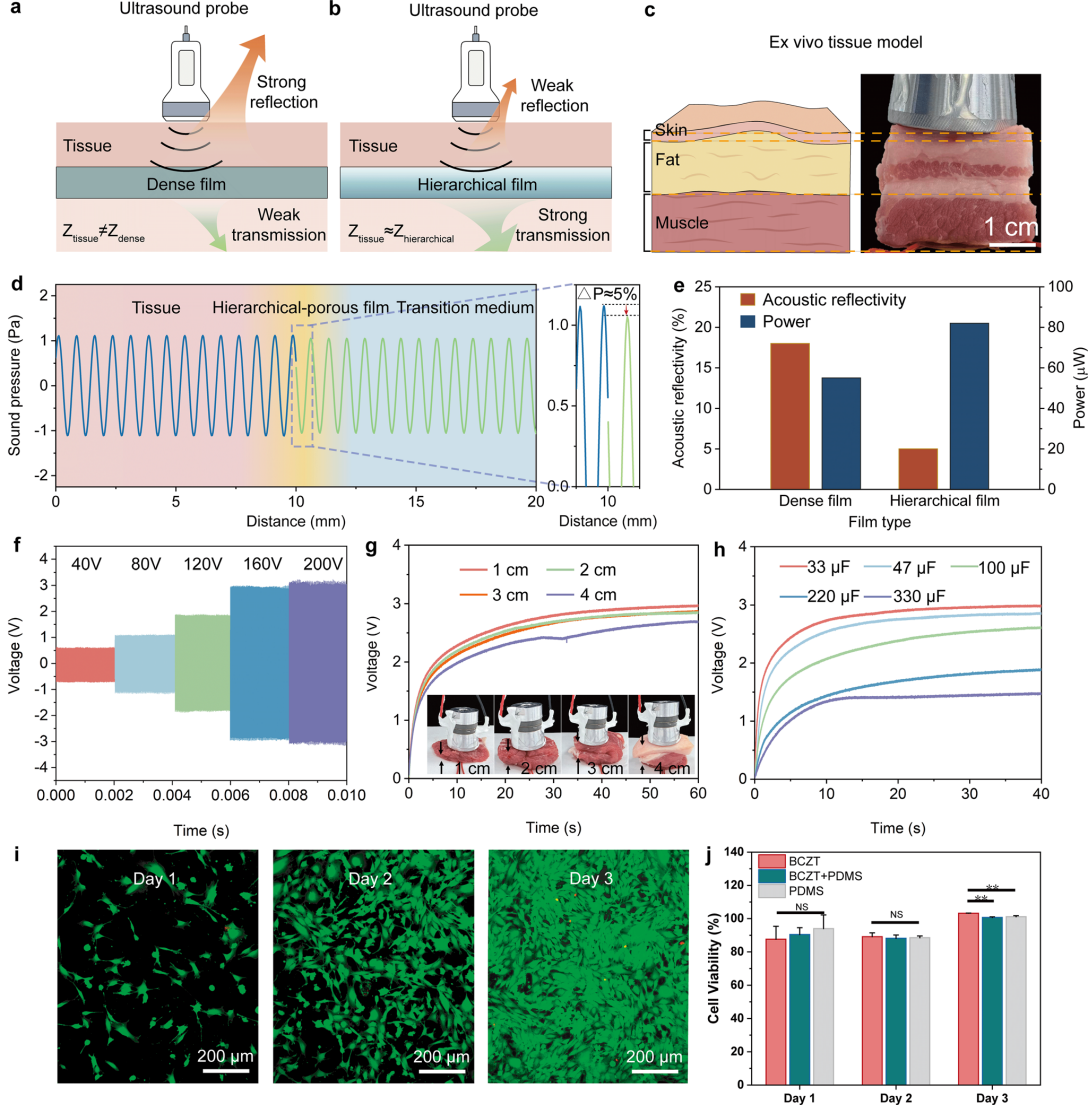
Tissue‐coupled ultrasonic energy harvesting, power delivery, and in vitro biocompatibility. (a, b) Schematics of acoustic reflection at the tissue interface for (a) dense and (b) hierarchical‐porous films. (c) Ex vivo porcine‐tissue model setup. (d) Acoustic‐pressure evolution along the propagation path and across the tissue‐film interface. (e) Simulated acoustic reflectivity and measured maximum output power of the two films. (f) Open‐circuit voltage under varying ultrasound driving voltages. (g) Charging curves of a 47 µF capacitor through varying tissue thicknesses. (h) Charging curves for different capacitors (33–330 µF) under a fixed 1 cm tissue thickness. (i) Representative live/dead fluorescence images of NIH‐3T3 fibroblasts cultured with BCZT extract on days 1‐3. Images are representative of three independent experiments; no statistical comparison was performed. (j) Quantitative viability of NIH‐3T3 fibroblasts cultured with extracts from BCZT, BCZT+PDMS, and PDMS samples over 3 days, assessed using a Cell Counting Kit‐8 (CCK‐8) assay. Data are presented as mean ± SD (n = 3 independent experiments). Statistical comparisons among groups at each time point were performed using one‐way ANOVA followed by Tukey's post hoc test. NS, *p* ≥ 0.05; ^**^ *p* < 0.01.

Based on the enhanced voltage sensitivity arising from the high g_33_ of the hierarchical‐porous BCZT film, its performance for tissue‐coupled ultrasonic energy harvesting was further evaluated. To assess the acoustic‐coupling process, the interaction between incident ultrasound and the tissue‐device interface was first examined. Dense and hierarchical‐porous films exhibited markedly different interactions with incident ultrasound in soft tissue. Because of the large acoustic impedance mismatch between dense piezoceramic films and biological tissue, a substantial fraction of the incident ultrasonic wave was reflected at the interface before reaching the active layer (Figure 4a). By contrast, the hierarchical‐porous film presented a lower effective acoustic impedance and a more compliant tissue‐contacting surface, which reduced the impedance discontinuity and thereby suppressed interfacial reflection (Figure 4b). As a result, ultrasound transmission into the active piezoelectric layer was enhanced, providing more effective acoustic input for electromechanical conversion.

To mimic realistic biological conditions, we established an ex vivo porcine‐tissue model for tissue‐mediated ultrasonic power transfer (Figure 4c). Given the compositional and structural similarities between porcine tissue and human soft tissue, this model provides a representative platform for evaluating acoustic propagation and device response under tissue‐relevant conditions. Acoustic‐field simulations were then performed to examine the evolution of acoustic pressure along the propagation path and across the tissue‐film interface (Figure 4d). The hierarchical‐porous film exhibited only a small reduction in transmitted pressure at the interface, indicating efficient acoustic transmission through the tissue‐coupled structure. By contrast, the dense BCZT film showed a much more pronounced pressure drop after transmission across the interface, and the transmitted acoustic pressure was markedly reduced, with an attenuation approximately three times larger than that of the hierarchical‐porous film under the same simulation conditions (Figure S14 and Note S4). This direct comparison confirms that the hierarchical‐porous architecture suppresses interfacial reflection and preserves a larger transmitted acoustic pressure, thereby enabling more effective acoustic energy coupling into the piezoelectric layer for ultrasonic energy harvesting.

Consistent with this trend, load‐resistance‐dependent output measurements were performed for both dense and hierarchical‐porous BCZT devices under the same ultrasound excitation condition. The output voltage increased with increasing external load resistance, whereas the output current decreased because of the larger resistance. As a result, the output power showed a clear maximum at an intermediate load resistance, corresponding to the optimal impedance‐matching condition (Figure S15). The dense‐film device delivered a maximum output power of approximately 55 µW at an optimal load resistance of 10 kΩ, while the hierarchical‐porous‐film device reached approximately 82 µW at an optimal load resistance of 47 kΩ. This comparison confirms that the hierarchical‐porous architecture improves the extractable electrical power under ultrasound excitation, rather than only increasing the open‐circuit voltage response.

To exclude the possibility that the enhanced output of the hierarchical‐porous device originates from accidental resonance near the operating frequency, impedance spectra of the dense and hierarchical‐porous BCZT devices were measured from 10 kHz to 1 MHz (Figure S16). Around the ultrasound operating frequency of 200 kHz, both devices show smooth variations in impedance magnitude and phase angle, without a pronounced impedance minimum, admittance peak, or abrupt phase transition. This result indicates that 200 kHz is not close to an electromechanical resonance of either device. Therefore, the improved output of the hierarchical‐porous device is attributed mainly to reduced dielectric permittivity and enhanced tissue‐device acoustic coupling, rather than resonance‐induced amplification.

The open‐circuit voltage response of the hierarchical‐porous BCZT device was then measured under a series of ultrasound driving voltages to evaluate its voltage‐generation behavior without external power extraction (Figure 4f). As the driving voltage increased, the open‐circuit voltage response increased correspondingly, demonstrating a clear dependence of the electrical response on the acoustic driving condition. This progressive increase confirms the robust acoustic‐to‐electrical responsiveness of the hierarchical‐porous BCZT film over the tested driving range.

To evaluate through‐tissue energy storage performance, capacitor‐charging measurements were further performed using the hierarchical‐porous device (Figure 4g,h). During capacitor charging, no external load resistor was connected; instead, the device output was connected to a half‐wave rectifier and then directly to the storage capacitor. Therefore, the capacitor acted as a time‐dependent capacitive load rather than a fixed resistive load. When the tissue thickness increased from 1 to 4 cm, the charging rate gradually decreased, which is consistent with ultrasound attenuation during propagation through tissue caused by reflection, scattering, and absorption (Figure 4g). The charging performance was also evaluated using storage capacitors ranging from 33 to 330 µF under a fixed tissue thickness of 1 cm (Figure 4h). Smaller capacitances charged more rapidly, while stable charging behavior was maintained across the entire capacitance range, demonstrating the capability of the device to operate with different energy‐storage capacitors.

To further evaluate the suitability of the hierarchical‐porous BCZT thin film for biointegrated and potential implantable applications, we assessed its in vitro cytocompatibility using NIH‐3T3 fibroblasts cultured with sample extracts (Figure S17). Live/dead fluorescence images revealed that the cells remained predominantly viable throughout the culture period, with increasing cell density from day 1 to day 3, indicating sustained cell proliferation in the presence of the extracts (Figure 4i). Consistent with this observation, quantitative cell‐viability measurements at days 1, 2, and 3 showed no obvious cytotoxicity in the tested extract groups during the tested culture period (Figure 4j). These results indicate that the hierarchical‐porous BCZT thin film exhibits acceptable cytocompatibility under the present extraction conditions, supporting its potential for wearable and implantable bioelectronics.

## 3 Conclusion

In conclusion, we developed freestanding hierarchical‐porous BCZT thin films for tissue‐coupled ultrasonic energy harvesting. The engineered porous‐to‐dense architecture enabled phase‐pure BCZT while preserving structural integrity and mechanical compliance. By redistributing bending‐induced tensile stress, the hierarchical‐porous structure suppressed crack initiation and improved the mechanical reliability of the freestanding film. Meanwhile, the reduced effective permittivity, together with retained piezoelectric activity, yielded a high piezoelectric voltage coefficient of g_33_ = 97 × 10^−3^ V·m/N. Under tissue‐relevant conditions, the hierarchical‐porous film showed reduced interfacial acoustic reflection, enhanced transmitted pressure, and improved acoustic‐to‐electrical energy conversion relative to the dense counterpart, resulting in an approximately 1.5‐fold enhancement in output power under optimized load conditions. These results establish hierarchical porosity as an effective strategy for simultaneously improving mechanical robustness, acoustic coupling, and electromechanical transduction in freestanding piezoceramic thin films, and highlight its potential for wearable sensing and implantable ultrasound‐powered bioelectronics.

## 4 Experiment

### 4.1 Preparation of Van Der Waals Epitaxial Layer

The mica substrates (purchased from Taiyuan Fluorophlogopite Mica Co., Ltd.) were cleaned sequentially with acetone, ethanol, and deionized water prior to film deposition. A thin Pt film (∼20 nm in thickness) was deposited on the mica surface via magnetron sputtering (Q150TS, QuorumTech) to serve as the van der Waals epitaxial growth layer.

### 4.2 Preparation of Hierarchical‐Porous BCZT Sol‐Gel Thin Films

BCZT sol was prepared using barium acetate, calcium acetate, zirconium n‐propoxide, and titanium (IV) butoxide as precursors, with poly (vinylpyrrolidone) (PVP, Mw ≈ 40 000) as the pore‐forming additive.

Barium acetate and calcium acetate (both Sigma‐Aldrich) were dissolved in glacial acetic acid and stirred at 100°C for 2 h to obtain Solution A. Titanium (IV) butoxide and zirconium n‐propoxide (both Sigma‐Aldrich) were dissolved in ethylene glycol monomethyl ether and stirred at 80°C for 2 h until a homogeneous pale‐yellow solution was formed, yielding Solution B. Solution B was then slowly added to Solution A under stirring at 80°C, and the resulting mixture was maintained at 80°C for 1 h with continuous stirring. The initial cation ratio in the mixed sol was fixed at Ba:Ca:Zr:Ti = 0.85:0.15:0.10:0.90. PVP was added to the BCZT sol, and the mixture was stirred at 80°C for 2 h until the PVP was fully dissolved, yielding a pale‐yellow sol.

Hierarchical‐porous BCZT thin films were fabricated on Pt‐coated mica substrates by spin coating. The precursor sol was dispensed onto the cleaned substrate and spun at 4000 rpm for 30 s. To construct a hierarchical‐porous architecture through the film thickness, a layer‐by‐layer deposition strategy was adopted in which the PVP content was progressively increased from the bottom layer to the top layer. After completing the layer‐by‐layer deposition, the PVP‐containing green films were subjected to a slow pre‐carbonization treatment under Ar at a heating rate of 1°C/min, with sequential holding steps at 300°C, 400°C, and 500°C for 2 h at each temperature. The pre‐carbonized films were then annealed in air at 800°C for 2 h to remove residual carbonaceous species and induce BCZT crystallization. The resulting hierarchical‐porous BCZT films had a total thickness of approximately 2.5 µm.

### 4.3 Characterization Method

Scanning electron microscopy images were acquired using a Regulus 8100 SEM. SEM‐EDS mapping was performed to analyze the exposed backside of the released BCZT/Pt film after water‐triggered lift‐off. The released film was transferred onto a Si wafer for SEM‐EDS characterization, and the mapped surface corresponded to the Pt‐side surface after release. Large‐area elemental mapping was conducted over a millimeter‐scale region to evaluate whether mica‐related residues remained on the released backside surface. The crystallinity of the BCZT films was characterized by X‐ray diffraction (XRD, Rigaku SmartLab). Piezoelectric properties were measured via piezoresponse force microscopy (PFM) using an Asylum Research MFP‐3D Infinity AFM. PFM switching spectroscopy was performed by applying a direct‐current (DC) bias sweep to the conductive AFM tip while recording the local phase and amplitude responses. The strain‐voltage response was obtained from the PFM amplitude signal to evaluate the local piezoelectric switching behavior. Young's modulus was determined using a Bruker TI980 nanoindentation system. Dielectric properties were measured using an inductance‐capacitance‐resistance (LCR) meter with a metal‐insulator‐metal capacitor configuration. Circular metal electrodes were deposited on the BCZT film surface as top electrodes, while the Pt layer served as the bottom electrode. The capacitance and dielectric loss were measured at 200 kHz. The relative permittivity was calculated according to ε_r_ = Ct/(ε_0_A), where C is the measured capacitance, t is the film thickness, ε_0_ is the vacuum permittivity, and A is the area of the circular top electrode.

### 4.4 Corona Poling of BCZT Thin Films

Before piezoelectric and ultrasonic‐output measurements, the BCZT thin films were polarized by corona poling. The freestanding BCZT/Pt film was placed on a flat stainless‐steel disk with a diameter of 5 cm, which served as the grounded bottom electrode. A corona voltage of 8 kV was applied at room temperature using a needle electrode positioned 10 mm above the film surface. The poling duration was 30 min. After poling, the samples were stored under ambient conditions for one day to reduce residual electrostatic interference before PFM, dielectric, and ultrasonic‐output measurements. The same poling conditions were used for both dense and hierarchical‐porous BCZT thin films.

### 4.5 Transfer of BCZT Films

Ethylene‐vinyl acetate (EVA) was dissolved in toluene to prepare a 0.1 g·mL^−1^ solution. The EVA solution was spin‐coated onto the BCZT surface at 1000 rpm for 30 s to form a support layer, followed by drying at 140°C for 10 min in air. To delaminate the film, one edge of the EVA/BCZT/Pt/mica stack was contacted with a water droplet, allowing capillary infiltration along the weak Pt‐mica interface. The EVA/BCZT/Pt layer detached from the mica and floated on the water surface. For transfer, the target substrate was submerged in water, and the floating film was gently guided onto it; the assembly was then retrieved and air‐dried to preserve the film integrity. Finally, the EVA support layer was removed by immersion in toluene, yielding the transferred BCZT film.

### 4.6 Phase‐Field Analysis of Fracture in Hierarchical‐Porous and Dense Ceramics

A phase‐field model [47, 48] was employed to investigate the fracture behavior of hierarchical‐porous and dense ceramics. The model was implemented in the finite element software ABAQUS via a user‐defined subroutine, and the constitutive formulation is provided in Note S2. A 2D plane strain model was established, with specimen dimensions of 10 µm in length and 2.5 µm in width. For the hierarchical‐porous ceramic, pores were distributed along the thickness direction with a hierarchical‐porous profile, with a porosity of 30 vol% and pore diameter ranging from 0.01 to 0.15 µm. In contrast, the dense ceramic contained no pores and served as the reference case. In both models, a triangular notch with a depth of 0.30 µm and a width of 0.06 µm was introduced at the top center of the specimen to trigger crack initiation. The damage state was described by a phase field variable ranging from 0 (intact) to 1 (fully damaged), and the material parameters are summarized in Table S1. A symmetric pure‐bending displacement‐controlled loading was applied. Reference points were defined at the midpoints of the two ends and kinematically coupled to the corresponding boundaries. During loading, equal but opposite rotational displacements were imposed at the two ends, with a maximum rotation angle of 0.004 rad, to drive crack initiation and final fracture. The mesh consisted of coupled triangular and quadrilateral elements, with a global mesh size of 0.005 µm, and local refinement was applied near the notch tip to accurately capture crack initiation and propagation. The phase field length scale parameter $l_{0}$ was set to 0.015 µm, which is more than twice the element size, ensuring the numerical accuracy and reliability of crack propagation prediction.

### 4.7 Device Fabrication

The EVA/hierarchical‐porous BCZT/Pt film stack was first transferred onto a 100 µm‐thick PDMS membrane screen‐printed with a 10 µm‐thick Ag layer. Residual EVA was removed with a small amount of toluene. A 10 µm‐thick Ag film with a 20 mm diameter was screen‐printed onto a second 50 µm‐thick PDMS membrane to enhance electrical signal intensity, and the Ag‐screen‐printed side was bonded to the BCZT film stack to complete the device encapsulation. To verify the practical applicability of the BCZT thin‐film sensor for physiological pulse signal monitoring, pulse‐wave tests were conducted on healthy volunteers.

### 4.8 Ultrasound Energy Harvesting Experiments

A focused ultrasound transducer with a nominal center frequency of 200 kHz was used as the acoustic source and driven by a function generator and a power amplifier. A 1–4 cm‐thick slice of fresh pork tissue was used as the pork tissue analog. The fabricated hierarchical‐porous BCZT device was affixed to one side of the tissue analog, with the transducer focus aligned with the device's active plane. To evaluate the voltage response of the device under ultrasound excitation, the driving voltage applied to the ultrasound transducer was varied stepwise, and the corresponding open‐circuit voltage response was recorded. The open‐circuit voltage was measured using a high‐impedance differential voltage probe with an input impedance of 10 MΩ, connected to a digital storage oscilloscope. Under this condition, the measurement was used to evaluate the voltage‐generation behavior of the device without external power extraction. To determine the extractable electrical power and the optimal load resistance, a series of external load resistors were connected to the device under the same ultrasound excitation condition. The root‐mean‐square (RMS) voltage across each load resistor was recorded, and the output power was calculated according to P_RMS_ = V_RMS_^2^/R, where V_RMS_ is the root‐mean‐square voltage across the load, and R is the load resistance. The load resistance giving the maximum output power was defined as the optimal load for the corresponding device. For alternating‐current‐to‐direct‐current (AC‐DC) rectification and electrical energy storage, a half‐wave rectifier circuit was connected to storage capacitors with capacitances of 33, 47, 100, 220, and 330 µF. No external load resistor was connected during the capacitor‐charging measurements. The capacitor voltage was monitored in real time using the digital storage oscilloscope.

### 4.9 In Vitro Biocompatibility

Fibroblast cell line NIH‐3T3 was purchased from the American Type Culture Collection (ATCC) and cultured in Dulbecco's modified Eagle's medium (DMEM, Gibco, # 11875085, USA) supplemented with 10% fetal bovine serum (FBS, Gibco, # 10270106, USA) and 1% penicillin‐streptomycin (Gibco, # 15140122, USA). The medium was changed every two days. Cells were cultured under 5% CO_2_ humidified atmosphere at 37°C and were passaged at 80% confluence.

BCZT, BCZT+PDMS, and PDMS samples were immersed into 75% ethanol overnight and rinsed with phosphate‐buffered saline (PBS). Then, the samples were exposed to ultraviolet (UV) light for complete sterilization. To obtain the extraction medium, the sterilized samples were immersed into DMEM for 24 h at 4°C, with the concentration of 0.1 g/mL, as instructed by GB/T 16886.12/ISO 10993‐12. The extract was filtered by 0.2 µm filter and stored at 4°C for further use.

The cells were seeded into the 96‐well plate at a density of 2×10^3^ with 100 µL DMEM. After overnight incubation, the DMEM was disposed of and was replaced by 100 µL of extraction medium. Cell viability was evaluated using the Cell Counting Kit‐8 (CCK‐8) assay on days 1, 2, and 3. The absorbance of the supernatant was measured at 450 nm. Meanwhile, the cells were stained with 5 µM Calcein AM (Invitrogen, #C3100MP, USA) under 37°C for 20 min and 5 µm Propidium Iodide (PI) (Sigma–Aldrich, #81845, USA) at room temperature for 1 min to identify the live and dead cells at each time point. The stained samples were imaged by a fluorescence microscope (Nikon, Eclipse Ci, Japan). All fluorescent images were processed by ImageJ software (National Institutes of Health, USA).

## 5 Ethics Statement and Statistical Analysis

This study involved non‐invasive human wrist pulse‐wave monitoring using a skin‐mounted wearable sensor. All procedures involving the human participant were conducted in accordance with the Declaration of Helsinki and were approved by the Research Construction and Development Department, The Hong Kong University of Science and Technology (Guangzhou) (Approval No. HKUST(GZ)‐HSP‐2026‐0060). Written informed consent was obtained from the participant prior to testing.

Unless otherwise stated, quantitative data are presented as mean ± standard deviation (SD). For each quantitative comparison, the sample size n is provided in the corresponding figure caption. No data were excluded unless explicitly stated. For electrical, dielectric, piezoelectric, mechanical, and ultrasonic‐output measurements, raw data were analyzed directly without transformation or normalization unless otherwise stated. When normalization was applied, the normalization method is described in the corresponding figure caption or Experimental Section.

Statistical comparisons between two groups were performed using a two‐sided Student's *t*‐test. For comparisons among more than two groups, one‐way analysis of variance (ANOVA) followed by Tukey's post hoc test was used. A significance level of α = 0.05 was used for all statistical analyses. Statistical significance was defined as NS, not significant; ^*^ *p* < 0.05; ^**^ *p* < 0.01; and ^***^ *p* < 0.001. Statistical analysis and graph plotting were performed using OriginPro.

## Author Contributions

Z.L. and S.L. conceived the project and designed the studies. Z.L., S.G., Y.C., Y.G., J.L., and S.L. performed experiments and analyzed the experimental data. W.H. and Y.C. worked on simulations. Z.L., X.W., C.T., Y.L., J.L., W.H., and S.L. wrote the manuscript. B.W., Y.Z., X.L., Y.H., and X.Y. reviewed and revised the manuscript. All authors discussed the results and commented on the manuscript. Z.L., S.G., W.H., and Y.C. contributed equally to this study and shared the first authorship.

## Conflicts of Interest

The authors declare no conflicts of interest.

## Supporting information




**Supporting File 1**: advs77409‐sup‐0001‐SuppMat.docx.

**Supporting File 2**: advs77409‐sup‐0002‐MovieS1.mp4.

## Data Availability

Raw data are available from the corresponding authors upon reasonable request.
