# Supplementary material for: Freestanding Hierarchical‐Porous BCZT Thin Films Enable Efficient Ultrasonic Energy Harvesting in Soft Tissue
Source: Adv Sci (Weinh). 2026 Aug 29:e77409. Online ahead of print. doi: 10.1002/advs.77409 (PMC13525412; doi:10.1002/advs.77409)
Supplement: Supplementary file 1 [file ADVS-9999-e77409-s001.docx]

Supplementary Materials for

• **Freestanding Hierarchical-Porous BCZT Thin Films Enable Efficient Ultrasonic Energy Harvesting in Soft Tissue**

Zifan Li^1†^, Shengwei Gao^1†^, Wang Hong^5†^, Yuxin Chen^1†^, Yiyang Gu^1^, Xu Wang^1^, Chen Tang^1^, Ying Hong^2^, Xuemu Li^6^, Xiaodong Yan^7^, Yi Zheng^6^, Yiming Liu^4*^, Junchen Liao^3*^, Biao Wang^2*^, Shiyuan Liu^1*^

*Corresponding author. Email: shiyuanl@hkust-gz.edu.cn.

**This PDF file includes:**

Supplementary Note 1 to 4

Supplementary Figure 1 to 17

Supplementary Table 1

**Other Supplementary Materials for this manuscript include the following:**

Supplementary Movie 1

**Supplementary Figures**

**Figure S1.** Large-area SEM-EDS analysis of the released BCZT/Pt film backside for delamination-plane verification. (a) SEM image and corresponding EDS elemental maps of the exposed backside of the released BCZT/Pt film transferred onto a Si wafer. (b) EDS sum spectrum collected from the large-area mapped backside region. (c) EDS elemental composition of the exposed backside region.

**Figure S2.** Photographic demonstration of two successive deposition-release cycles using the same mica substrate

**Figure S3.** Surface integrity of the BCZT film after EVA removal. (a) AFM height image of the BCZT film surface after EVA removal. (b) Three-dimensional AFM topography of the same scanned region. (c) Large-area SEM image of the BCZT film after EVA removal.

**Figure S4.** Temperature-time profile for PVP pre-carbonization of the BCZT green film under Ar atmosphere. The as-deposited PVP-containing BCZT film was heated at a slow ramping rate of 1 °C min^-^¹ and held at 300, 400, and 500 °C for 2 h at each stage to gradually carbonize the PVP phase before oxidative annealing.

**Figure S5.** Temperature-time profiles for subsequent air annealing of the pre-carbonized hierarchical-porous BCZT films. After pre-carbonization, the films were annealed in air to promote the removal of carbonized organics and the crystallization of the BCZT phase. Final annealing temperatures of 700, 800, and 900 °C with a dwelling time of 2 h were used for comparison.

**Figure S6.** XRD patterns of hierarchical-porous BCZT films annealed at different temperatures. The film annealed at 700 °C shows insufficient crystallization and a residual carbon-related peak. In contrast, the films annealed at 800 and 900 °C exhibit well-defined diffraction peaks corresponding to the BCZT phase, indicating successful crystallization at higher annealing temperatures.

**Figure S7.** Cross-sectional SEM images of hierarchical-porous BCZT films annealed at 800 and 900 °C. (a) At 800 °C, the film exhibits a well-preserved porous microstructure with abundant pores across the thickness. (b) At 900 °C, partial pore collapse is observed, which is attributed to excessive sintering and the resulting densification of the porous network.

**Figure S8.** Surface and cross-sectional SEM images of BCZT films prepared with different PVP contents. (a-d) Surface SEM images of films prepared with 5, 10, 15, and 20 wt% PVP, respectively. (e-h) Corresponding cross-sectional SEM images. As the PVP content increases, the pore fraction gradually increases and the pore structure evolves from isolated pores to an interconnected porous network.

**Supporting Note 1**

**Finite-element mechanical simulation framework**

Finite-element simulations were performed in ABAQUS using a simplified layered model to analyze the mechanical behavior of the flexible device under bending and twisting deformations. The device was modeled as a PDMS/BCZT/PDMS sandwich structure. The BCZT film had a total thickness of 2.5 μm and was simplified as a three-layer structure to reflect the modulus gradient of the hierarchical-porous ceramic, with Young’s modulus of 130, 90, and 70 GPa, respectively, obtained from nanoindentation measurements. The Poisson’s ratio of the BCZT layers was set to 0.3 according to literature values. Owing to their negligible thicknesses, the Pt and Ag electrode layers were omitted from the mechanical model. Two opposite edges of the device were fixed during simulation. Bending loads and twisting loads were applied with a bending angle of ~50° and a twisting angle of 15°, respectively. The von Mises stress distribution was used to evaluate the mechanical response of the device.

**Figure S9.** Mechanical deformability of the flexible pulse-wave sensor based on the hierarchical-porous BCZT film. (a,b) Photographs of the device under bending and twisting deformation. Corresponding finite-element simulations show the stress distribution in the device under the two loading modes, indicating a relatively distributed stress state during deformation.

**Supporting Note 2**

**Phase field model framework**

The fracture behavior was modeled within a phase field framework implemented in ABAQUS through a user-defined subroutine. The formulation follows the variational theory of brittle fracture, in which the total potential energy consists of the bulk elastic energy and the fracture surface energy. In the phase field method, the total potential energy consists of the bulk elastic energy $W^{b}$ and the fracture surface energy $W^{s}$:

$$W=W^{b}+W^{s}.$$

The surface energy is the product of the critical energy release rate and the crack surface $A$ and is given by:

where $\gamma$ is the regularized crack surface density function that diffused in the bulk material, and given as:

where represents the phase field, 0 and 1 denote the intact and the fully damaged material, respectively. is a parameter that controls the diffusion width of the crack ‘‘length scale”. The bulk energy can be expressed in terms of the phase field and strain tensor:

where the elastic energy density is then decomposed into tensile ($\psi_{b}^{+}$) and compressive ($\psi_{b}^{-}$) parts, and the degradation function only acts on the tensile energy density $\psi_{b}^{+}$. A spectral decomposition scheme of the elastic energy density recommended by Miehe et al. is specified by

$$\psi_{b}^{\pm}=\lambda\langle\mathrm{tr}(\hat{\varepsilon})\rangle_{\pm}^{2}/2+G\cdot\mathrm{tr}\left(\langle\hat{\varepsilon}\rangle_{\pm}^{2}\right),$$

where $\lambda$ and $G$ are Lame constants, $\hat{\varepsilon}$ is the vector of principal strains. To describe quasi-brittle fracture, the constitutive response was formulated using a PF-CZM degradation law:

where $p=2$ is a shape parameter and, and critical fracture energy density $\psi_{c}$ is written as:

$$\psi_{c}=\frac{\sigma_{c}^{2}}{2E},$$

where $\sigma_{c}$ is critical tensile strength and $E$ is Young’s modulus. An efficient and robust staggered solution scheme was adopted, in which the displacement field and the phase field were solved in a decoupled manner. The simulations were performed under plane strain conditions. The phase field length scale was set to satisfy the mesh resolution requirement and was chosen to be larger than twice the characteristic element size, ensuring sufficient accuracy in crack initiation and propagation predictions.

**Table S1. Material properties of ceramic material.**

| Property | Ceramic material |
| --- | --- |
| Tensile modulus $E$ (GPa) | 130 |
| Poisson’s ratio $v$ | 0.3 |
| Strength $\sigma_{c}$ (MPa) | 49.78 |
| Critical energy release rate  (N/m) | 5.64 |

To enable quantitative comparison of fracture toughness in conventional stress-strain terms, the reaction moment $M$ and end-rotation angle $\theta$ obtained from the simulations were converted into equivalent (nominal) flexural stress and strain based on classical Euler-Bernoulli beam theory. Under the applied symmetric pure-bending condition, the specimen deforms into a circular arc; assuming the plane-sections-remain-plane hypothesis, the equivalent strain at the outer fiber is given by

$$\begin{matrix} & \varepsilon=\frac{\theta t}{2L} & & \text{(8)} \end{matrix}$$

where $t$ is the specimen thickness (the dimension along which stress and strain vary linearly) and $L$is the span of the pure-bending region between the two loading reference points. Correspondingly, assuming a linear elastic stress distribution across the cross-section, the equivalent flexural stress is expressed as

$$\begin{matrix} & \sigma=\frac{6M}{bt^{2}} & & \text{(9)} \end{matrix}$$

where $b$ is the out-of-plane thickness of the specimen. Equation (9) follows from the classical flexure formula $\sigma=My/I$, evaluated at the outer surface ($y=t/2$) for a rectangular cross-section with second moment of area $I=bt^{3}/12$. The fracture toughness of each specimen was then quantified as the area enclosed under the resulting equivalent stress-strain curve up to the point of final fracture. It should be noted that $\varepsilon$and $\sigma$obtained in this manner represent homogenized, nominal quantities; the true local stress and strain fields around individual pores and the notch tip deviate from this linear approximation and are instead explicitly resolved by the phase-field simulations described above. The corresponding toughness of each specimen, defined as the area under the equivalent stress-strain curve up to the point of final fracture, was calculated as

$$\begin{matrix} & U_{f}=\int_{0}^{\varepsilon_{f}} \sigma\text{ }d\varepsilon& & \text{(10)} \end{matrix}$$

which represents the energy absorbed per unit volume during the fracture process. This quantity was used to compare the energy dissipation capability of the hierarchical-porous and dense ceramics.

**Figure S10.** Evolution of the damage field in (a) hierarchical-porous and (b) dense ceramics under symmetric bending.

**Figure S11.** Comparison of the microstructure and piezoresponse of hierarchical-porous and dense BCZT thin films. (a,b) Cross-sectional and surface SEM images of the hierarchical-porous BCZT thin film. (c,d) Cross-sectional and surface SEM images of the dense BCZT thin film. (e) PFM amplitude image of the dense BCZT thin film. (f) PFM amplitude as a function of AC voltage, showing the piezoresponse behavior of the dense BCZT thin film.

**Figure S12.** Local ferroelectric and piezoelectric switching responses of dense and hierarchical-porous BCZT thin films measured by PFM. (a) PFM phase-voltage and strain-voltage responses of the dense BCZT thin film. (b) PFM phase-voltage and strain-voltage responses of the hierarchical-porous BCZT thin film.

**Supporting Note 3**

**Equivalent electrostatic simulation of the poling-field distribution in dense and hierarchical-porous BCZT films**

The simulations were performed using the Electrostatics interface in COMSOL Multiphysics. A stationary two-dimensional cross-sectional model was used. The dense and hierarchical-porous films had identical external dimensions of 10 μm×2.5 μm, corresponding to the representative in-plane width and experimental film thickness, respectively. The dense film was modeled as a homogeneous BCZT rectangle. The porous film contained closed air domains distributed in three pore-size levels through the thickness to reproduce the experimentally observed hierarchical morphology. The pore geometry was constructed from the cross-sectional microstructure and adjusted to a two-dimensional pore-area fraction of approximately 30 vol%. The pore area was included explicitly; therefore, the ceramic matrix was assigned the dielectric constant of dense BCZT rather than the effective dielectric constant of the porous film.

Because the air pores were explicitly included as independent computational domains, the ceramic matrix was assigned the experimentally measured relative permittivity of the dense BCZT film, ε_r_=560, rather than the effective permittivity of the porous film. The relative permittivity of the air pores was set to 1. Continuity of electric potential and normal electric displacement across the BCZT-air interfaces was automatically imposed by the electrostatic formulation.

The bottom boundary of each film was grounded (V=0), representing the grounded bottom electrode. An equivalent potential of 6.5 V was applied to the upper boundary. This potential difference represents the effective field across the film after corona-induced surface charging. The lateral external boundaries were electrically insulated, and no additional boundary condition was imposed at the internal BCZT-air interfaces.

**Figure S13.** Simulated electric-field magnitude distributions in dense and hierarchical-porous BCZT films under equivalent poling conditions.

**Supporting Note 4**

**One-dimensional simulation of acoustic-pressure transmission through dense BCZT film**

A one-dimensional finite-element model was established in COMSOL Multiphysics to simulate the propagation of an acoustic wave through tissue and a dense BCZT thin film. In the model, the distance axis represents the acoustic propagation direction. The tissue region was approximated as water, and the dense BCZT film thickness was set to 2.5 μm. The material parameters of dense BCZT were defined as follows: density = 5.86 g cm^-^³, Young’s modulus = 130 GPa, Poisson’s ratio = 0.3, and sound velocity = 2600 m s^-^¹. A continuous sinusoidal acoustic wave with an initial pressure amplitude of 1.1 Pa was applied as the incident excitation. The spatial evolution of acoustic pressure along the propagation direction was extracted to evaluate pressure transmission across the tissue/film interface. The pressure attenuation was quantified according to

$$\Delta P=\frac{P_{incident}-P_{transmitted}}{P_{incident}}$$

where $P_{incident}$ and $P_{transmitted}$ denote the incident and transmitted pressure amplitudes, respectively. The simulation result indicates that the transmitted pressure decreases markedly after passing through the dense BCZT film, reflecting the strong acoustic mismatch of the dense ceramic layer.

**Figure S14.** Simulated acoustic-pressure evolution across the tissue/dense-BCZT interface. The transmitted acoustic pressure after the dense film is markedly reduced, with an attenuation approximately three times larger than that of the porous film under the same simulation conditions.

**Figure S15.** Load-resistance-dependent output performance of dense and hierarchical-porous BCZT devices under ultrasound excitation. (a) Output voltage, current, and power of the dense BCZT device as functions of external load resistance. (b) Output voltage, current, and power of the hierarchical-porous BCZT device as functions of external load resistance.

**Figure S16.** Impedance spectra of dense and hierarchical-porous BCZT thin-film devices. (a) Frequency-dependent impedance magnitude ∣Z∣ and phase angle θ_z_ of the dense BCZT device. (b) Frequency-dependent impedance magnitude ∣Z∣ and phase angle θ_z_ of the hierarchical-porous BCZT device.

**Figure S17.** Live/dead fluorescence images of NIH-3T3 fibroblasts cultured with extracts from BCZT, PDMS+BCZT, PDMS, and control medium on days 1, 2, and 3. Live cells are shown in green and dead cells in red. All groups maintained predominantly live cells with very limited dead cells during the 3-day culture period. The increasing cell density from day 1 to day 3 further indicates normal cell proliferation. No obvious cytotoxicity was observed for the BCZT-containing samples. Representative images are from n = 3 independent experiments; no statistical comparison was performed.
